# Destination integration: linking physiology, histology, and embryology content in foundational sciences

**DOI:** 10.3389/fphys.2023.1236562

**Published:** 2023-09-14

**Authors:** Jennifer F. Dennis, Bradley A. Creamer

**Affiliations:** ^1^ Department of Pathology and Anatomical Sciences, Kansas City University, Kansas City, MO, United States; ^2^ Department of Basic Sciences, Kansas City University, Joplin, MO, United States

**Keywords:** physiology, integration, histology, embryology, female reproductive system

## Abstract

Anatomy and physiology are tightly linked disciplines that complement each other, however, in medical education delivery of this content is often siloed and divided. To address this, we created combined anatomy and physiology content for the female reproductive system, and team-taught designated histology and embryology topics integrated with the physiology content. Collectively, this created a more holistic incorporation of topics for student learning. Here we describe the format and approach for this teaching innovation.

## 1 Introduction

The first-year medical curriculum in College of Osteopathic Medicine (COM) at Kansas City University is delivered using a two-pass, modified systems-integrated approach. Courses in the first year that incorporate physiology as well as anatomy, including its subdisciplines of histology and embryology, are the Musculoskeletal I (MSK), Neuroendocrine (NEER), Cardiopulmonary-Renal I (CPR), and Gastrointestinal-Genitourinary (GIGU) courses; this includes 92.5 anatomy lecture hours with each subdiscipline represented by the following: gross anatomy, 58.5 h; histology, 17 h; embryology, 17 h. To complement the lecture content, a total of 68 h in the gross anatomy laboratory are included within the Musculoskeletal, Neuroendocrine, Cardiopulmonary and Renal I, and Gastrointestinal-Genitourinary courses. The physiology content for these courses is comprised of a total of 80 lecture hours within MSK, NEER, CPR and GIGU. Assessments in these anatomy-based courses include both written and laboratory-based practical examinations. Additional courses in the first year of study include Scientific Foundations of Medicine, Essentials of Clinical and Osteopathic Skills, I and II, Bioethics I, Medical Informatics I and II, and Mechanisms of Disease; these courses do not have anatomy- or physiology-specific content. Within the KCU curriculum, systems-based curricula are developed using Backwards Course Design (Wiggins and McTighe, 2005), which involves designing a course from the end (i.e., assessments of competency) of the learning process to the beginning. In this process, faculty initially evaluate what they want their students to learn and/or achieve while taking their course, followed by the teaching faculty developing learning goals for the course. Ideally these course goals consider the long-term integration of content, while facilitating an intuitive integration of course activities relevant to the topics covered. Historically, teaching faculty create learning objectives (outcomes), specific to their teaching content, but also independent of each other’s disciplines. Additionally, faculty prepare a designated number of assessment items to be incorporated on summative, multiple-choice written exams.

Think-pair-share (TPS) is a cooperative learning technique utilizing three steps to encourage and engage student discussion of educational concepts Lyman 1981. The technique includes three steps: 1) students think about a given question or problem and organize their thoughts, ideas, and answers to the given questions; 2) students work in pairs to discuss their answers; and 3) students share their ideas with the whole group (Lyman and Anderson, 1981). The second and third steps are fundamental to the cooperative learning process as they each give students the opportunity to think and identify what they may or may not know about a concept, as well as confirm in a paired, lower-stake conversation what is correct and/or incorrect about their initial ideas. Overall, the think-pare-share technique creates a learning environment that incorporates interactive discussion where students can reflect on their own ideas as well as their peers (Pluta et al., 2013; Smith, 2016; Carpenter et al., 2020; Cooper et al., 2021). A frequent utilization of the think-pair-share strategy is best characterized as component of the “flipped classroom,” where TPS is incorporated for learners to review content materials before the teaching session, to then pair up and share concepts, ideas, and/or answers to questions posed by the educator. Indeed, positive outcomes incorporating TPS within flipped classrooms have been documented for a range healthcare professional programs including medicine (Rao and DiCarlo, 2000; Pluta et al., 2013; Moffett, 2015; Carpenter et al., 2020), dentistry (Allen et al., 2022), pharmacy (McLaughlin et al., 2014), and physician assistant (Deshpande et al., 2020), as well residency program (Martinelli et al., 2017) education. While there is rich evidence of incorporating the technique with students, few reports have described using this format with educators themselves. Indeed, a recent report described utilizing TPS in engaging curriculum discussions with dental educators in a professional development retreat (Ramesh et al., 2021), but we have not identified any that describe utilizing of the TPS technique in curriculum development.

**FIGURE 1:**
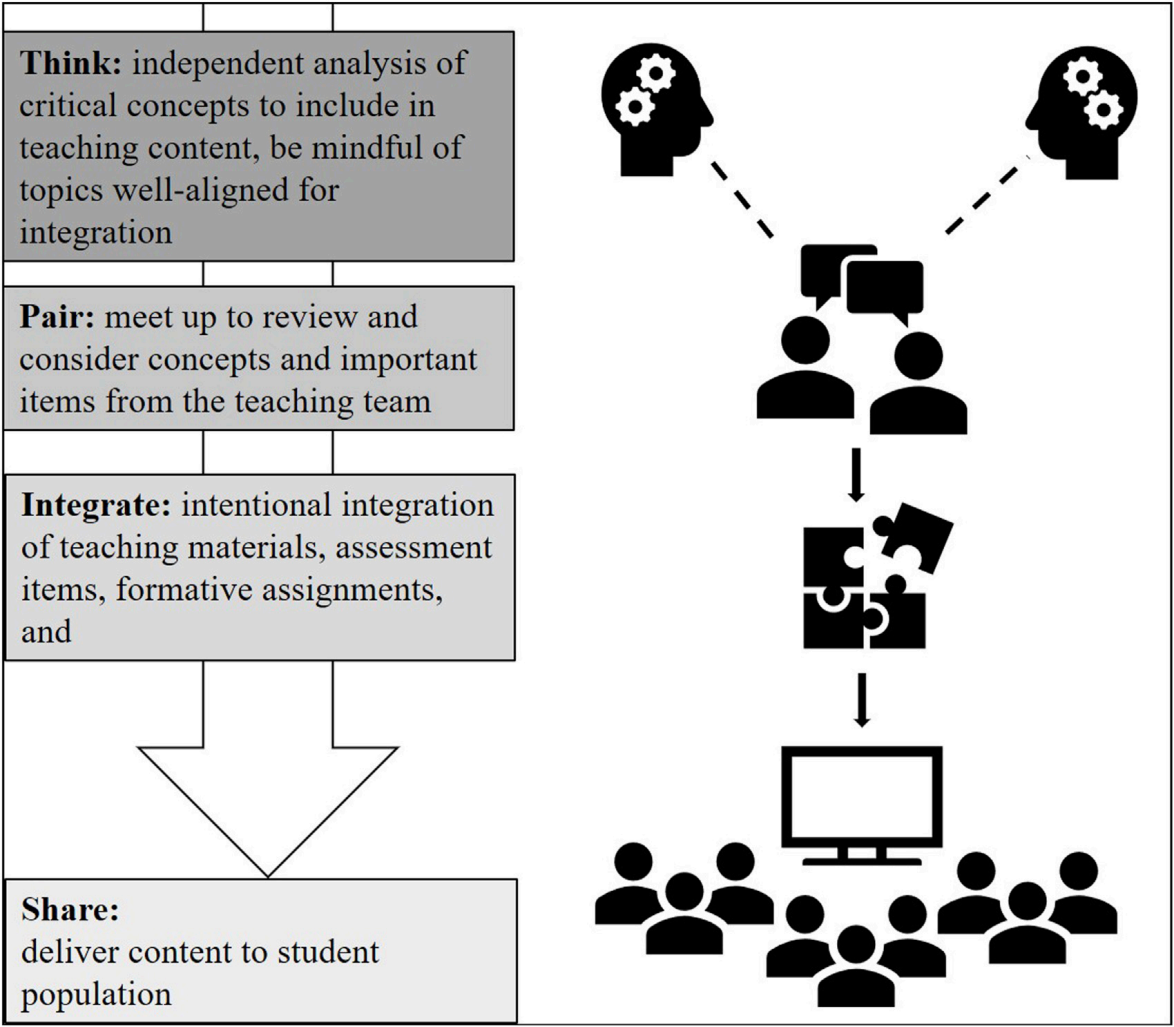
Operational Structure of the ‘Think-Pair-Integrate-Share’ Format.

Here we describe an expansion of the TPS technique relevant to integrating multiple content disciplines. Our innovative “Think-Pair-Integrate-Share” (TPIS) approach was utilized to identify and integrate independent physiology and histology content into a team-taught, integrated format (Table 1). Due to frequent and overlapping nomenclature and concepts, we identified multiple content areas within the female reproductive system as key teaching areas where a reorganization of select physiology, histology and embryology topics would better serve the content delivery and likely improve student success. In total, seven content areas were integrated using the “Think-Pair-Integrate-Share” technique (Figure 1), resulting in more comprehensive and holistic teaching materials being distributed to students.

**TABLE 1:** Examples of integrated and expanded concepts.

Lecture	Content discipline	Learning objectives (Outcomes)
Gynecology: Menstrual Cycle and Menarche	Physiology, Histology	In order to explain amenorrhea due to polycystic ovarian syndrome, describe and identify the primordial follicle, primary follicle, secondary follicle, and mature (Graafian) follicle on histological images, including cell types, functions, features, and/or layers
		In order to explain bleeding in pregnancy, describe and identify the corpus luteum and corpus albicans on histological images, including any distinguishing features
		In order to explain female infertility, you should be able to describe the hormone synthesis and secretion changes within the dominant follicle during the periovulatory period
		In order to explain female infertility, you should be able to describe ovulation, and the process of luteinization on the theca and granulosa cells, and the roles of hormones in each of these processes
Parturition and Lactation	Physiology, Histology	In order to explain a congenital breast malformation, such as supernumerary nipple, you should be able to describe the histology of the mammary gland and structural changes that occur during puberty, pregnancy, and lactation
		In order to explain breast discharge, you should be able to describe the hormonal regulation of mammary gland development (i.e. puberty vs pregnancy)
		In order to explain breast discharge, you should be able to describe the five major pathways of secretion of milk components by alveolar cells
Pregnancy and the Fetus	Physiology, Embryology	In order to describe a patient presentation of small for gestational age due to maternal lifestyle, describe the placental membrane and differentiate between substances that can or cannot cross the placenta
		In order to explain Intrauterine growth restriction, you should be able to describe how the maternal-placental-etal unit is involved in hormone synthesis during pregnancy
		In order to explain Intrauterine growth restriction, you should be able to describe the maternal serum levels and the physiological functions of maternal-fetal-and placental-derived hormones

## 2 Materials and methods

**FIGURE 2:**
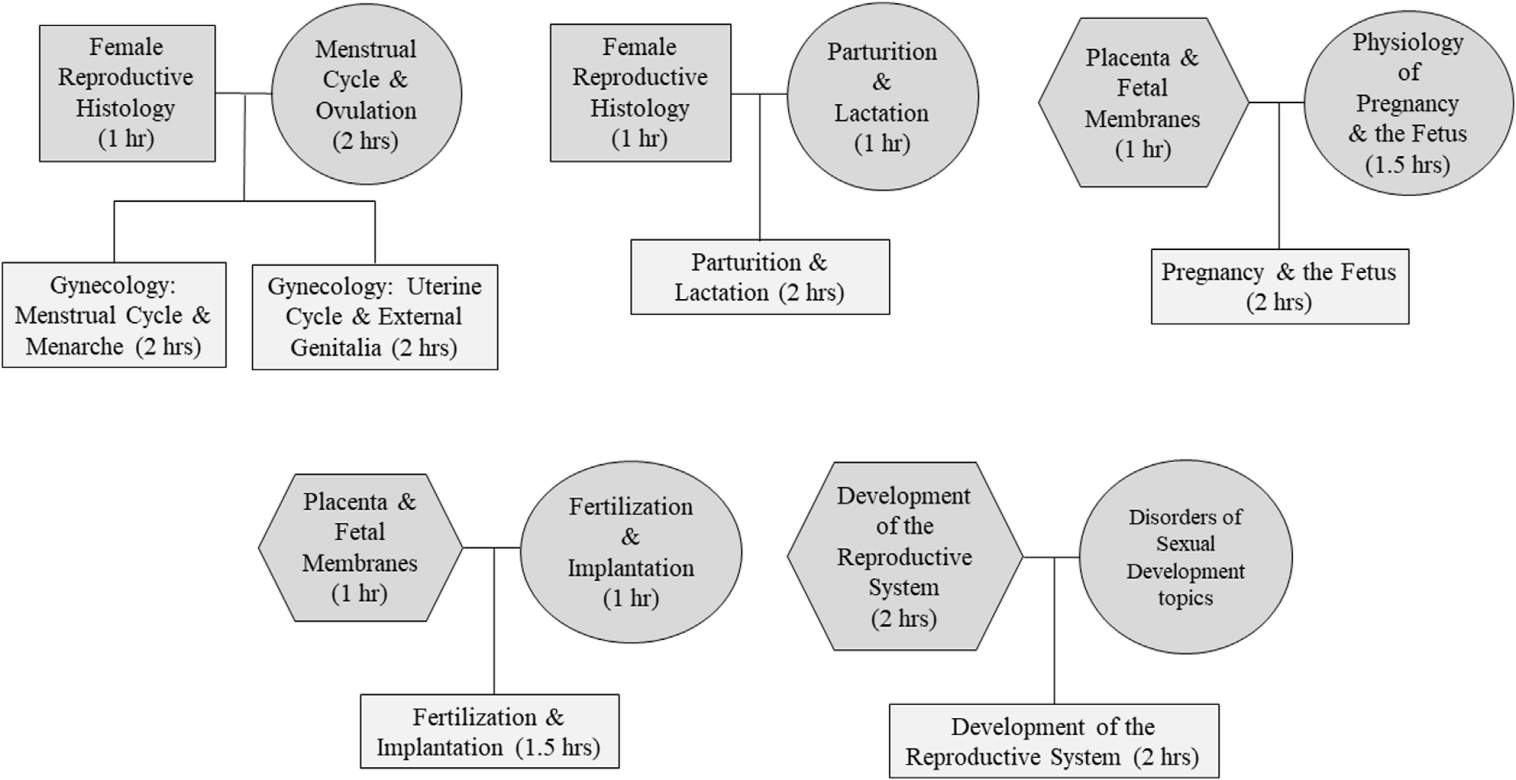
Pedigree chart for lecture content integrated with the ‘Think-Pair-Integrate-Share’ Format.’ Represented disciplines include Physiology = circles, Anatomy = squares, Embryology = hexagon, and Integrated = rectangles.

A total of 8.5 h of female reproductive content was identified for the think-pair-integrate-share format. This included one histology female reproductive histology (1 h) and two embryology lectures, development of the reproductive system (1 h) and placenta and fetal membranes (1 h). The physiology lectures selected for integration included: menstrual cycle and ovulation (2 h), fertilization and implantation (1 h), physiology of pregnancy and the fetus (1.5 h), parturition and lactation (1 h). The reorganization and integration of topics resulted in the generation of the following lectures: gynecology: menstrual cycle and menarche (2 h), gynecology: uterine cycle and external genitalia (2 h), fertilization and implantation (1.5 h), development of the reproductive system (2 h), pregnancy and the fetus (2 h), and parturition and lactation (2 h) (Figure 2).

To incorporate the think-pair-share integration, the authors utilized the backwards design template and course goals generated by the course director to develop independent teaching elements, such as learning objectives (outcomes), written examination questions, and teaching materials. With the backwards design framework established, the teaching faculty (BAC and JFD) independently developed each updated sets of learning objectives (outcomes) from those used in the previous course iteration (2021–2022 academic year). This permitted each to identify critical topics that were preferential to include, which were then shared with the other co-instructor/author. Subsequently, the authors paired up, in-person, to review each set of learning objectives to identify redundancies, content/topic gaps, and finalize the order for presentation to the students (Figure 1). Written examination questions and teaching materials were finalized in a manner similar to the learning objectives; faculty worked on each set of items individually, paired up, and then integrated the items from each content discipline.

To “share” the integrated content, lectures were delivered either asynchronously using pre-recorded mp4 videos or synchronously via live, in-person lectures. Asynchronous lectures involved each faculty member recording their lecture video in an mp4 format using Movavi Video Software (Movavi Software Inc., Wildwood, MO, United States). The videos were “paired” by BAC and combined into a single, integrated video that was subsequently “shared” with the students via the course learning management system (Canvas, Instructure, Salt Lake City, UT, United States). For lectures delivered synchronously and in-person, the content slides were prepared by individual instructors (BAC, JFD) and “paired” into a single PowerPoint presentation (Microsoft Corporation, Redmond WA, United States). The slides were “shared” with the students with each faculty presenting their specific content discipline; both faculty were available for student questions during and immediately following the teaching sessions.

The teaching materials also included a set of higher-order, board examination style questions for each lecture that were developed using the same “think-pair-integrate-share” technique. The questions required synthesis and evaluation of the teaching material; correct answers and rationales were included for each of the questions. The questions covered both teaching disciplines and served as a formative assessment provided in an electronic format through our learning management system.

## 3 Results

### 3.1 What worked well

The think-pair-integrate-share technique was an effective mechanism for updating and integrating physiology and histology or physiology and embryology concepts into a single, comprehensive format. Independently evaluating teaching content, followed by pairing up with a teaching colleague to review and integrate content disciplines was an innovative method for updating and combining information. The feedback and discussions generated were helpful in ensuring one’s foundational and motivation for including topics and/or concepts, as well as vetting examination questions, lecture slides, and learning objectives. Future goals include the evaluation of assessment data to complete a meaningful analysis of student performance outcomes from before and after content integration. Similarly, incorporating supplemental questions in future course evaluations to evaluate student perceptions of the integrated content as the end-users of the updated format.

### 3.2 What may not work as well

As a concept itself, successful integration depends on individual topics or items being somewhat relevant to each other. This was fundamental to the topics chosen specific to the female reproductive system that were described here. The “think-pair-integrate-share” approach would likely not be as effective if the pairing up of information included topics that were not as immediately relevant to each other. An additional hurdle could be the working relationship of teaching faculty hoping to think-pair-integrate-share; the success of this endeavor was directly linked to the authors having a mutually beneficial professional relationship, and we could each identify the overlap in concepts that could be used to our benefit. This would not have been as successful if either individual were less open to sharing course and teaching materials, including exam questions, and giving up dedicated teaching time in front of students. Finally, it was helpful that the authors have a similar teaching style; it would be difficult to integrate and share two vastly different instructional approaches. While not impossible, we believe it would be more beneficial to utilized a single instructional method to ensure teaching continuity in a single lecture.

### 3.3 Lessons learned

Utilizing the TPIS technique is a viable and holistic approach to integrating course content from different, yet interrelated disciplines. In considering recommendations for others in successfully adopting the TPIS format to blend teaching materials, we noted the following: 1) identify relevant, and interdependent topics/concepts for integration across disciplines of interest; 2) provide ample time for the TPIS technique–time to independently “think” on and assess one’s individual topics and time to “pair” and “integrat”integrate concepts across disciplines successfully, these should be considered months in advance, and begin with the backwards design process. Finally, consider the audience to whom the integrated concepts will be “shared” concepts “integrated” (Pluta et al., 2013) and “shared” should be relevant and important to the audience to enhance engagement and technique success.

## 4 Conclusion

Historically at KCU, histology, embryology, and physiology lectures specific to the female reproductive system were developed, delivered and assessed independently of each other. The incorporation of the TPIS technique resulted the integration of multiple concepts classically associated with different content disciplines. Indeed, faculty members pairing up and integrating the content resulted in a holistic representation of histology and physiology, or embryology and physiology concepts. Future efforts are directed at evaluating the student experience utilizing the integrated teaching materials and their perceptions of the teaching activities. This feedback will be utilized to quantitively evaluate the TPIS technique, with the goal of applying the approach to other closely related concepts in histology and physiology, or embryology and physiology teachings. Characterizing the TPIS technique will further characterize and enhance curricular content, and may encourage other programs to adopt the updated technique.

## Data Availability

The original contributions presented in the study are included in the article/Supplementary material, further inquiries can be directed to the corresponding author.
